# Strong and highly asymmetrical optical absorption in conformal metal-semiconductor-metal grating system for plasmonic hot-electron photodetection application

**DOI:** 10.1038/srep14304

**Published:** 2015-09-21

**Authors:** Kai Wu, Yaohui Zhan, Cheng Zhang, Shaolong Wu, Xiaofeng Li

**Affiliations:** 1 College of Physics, Optoelectronics and Energy & Collaborative Innovation Center of Suzhou Nano Science and Technology, Soochow University, Suzhou 215006, China; 2 Key Lab of Advanced Optical Manufacturing Technologies of Jiangsu Province & Key Lab of Modern Optical Technologies of Education Ministry of China, Soochow University, Suzhou 215006, China

## Abstract

We propose an architecture of conformal metal-semiconductor-metal (MSM) device for hot-electron photodetection by asymmetrical alignment of the semiconductor barrier relative to the Fermi level of metals and strong energy localization through plasmonic resonances. Compared with the conventional grating design, the multi-layered grating system under conformal configuration is demonstrated to possess both optical and electrical advantages for high-sensitivity hot-electron photodetection. Finite-element simulation reveals that a strong and highly asymmetrical optical absorption (top metal absorption >99%) can be realized under such a conformal arrangement. An analytical probability-based electrical simulation verifies the strong unidirectional photocurrent, by taking advantage of the extremely high net absorption and a low metal/semiconductor barrier height, and predicts that the corresponding photoresponsivity can be ~3 times of that based on the conventional grating design in metal-insulator-metal (MIM) configuration.

Nanostructured metals have received extensive recent attention due to the strong energy localization under the unique photonic properties^1^, light concentration^2^, substantially improved optical absorption^3^, etc., by exciting surface plasmons (SPs). SPs can find a variety of applications, such as perfect absorbers^4^,^5^, photothermal therapy^6^, biosensing^4^,^7^,^8^,^9^, nonlinear optics^10^,^11^, photocatalysis^12^,^13^, and photovoltaics^14^,^15^,^16^. Especially, photodetection and photovoltaic conversion based on plasmonic hot electrons have been widely discussed in recent years^17^,^18^,^19^,^20^,^21^,^22^,^23^,^24^. The most frequently discussed systems are based on the metal-semiconductor (MS) configuration, where the photocurrent is generated from those electrons that can conquer the Schottky barrier on the MS interface^17^,^20^. Based on different mechanisms, however, it was demonstrated that the hot electrons induced by non-radiative decay of SPs can also be collected via metal-insulator-metal (MIM) configuration^18^,^19^,^21^. However, a key flaw of such system lies in that the hot electrons excited by low-frequency photons can hardly conquer the relatively high metal-insulator barrier, leading to low photoresponsivity^18^,^21^. The low responsivity is the result of a number of factors, including imperfect optical absorption and unoptimized electrical transport.

For hot-electron photodetection, a high and unidirectional photocurrent is strongly desired. However, in the considered MIM systems, both the top and the bottom metallic layers absorb the incident energy, but they generate reverse photocurrents unfortunately. This reveals that the total absorption of the entire system is not an ultimate target; nevertheless, a strong and asymmetrical absorption is needed eventually, i.e., highly distinguished absorption between the top and the bottom metallic layers. However, the existing systems can hardly fulfill this stringent requirement since the incident light couples strongly with both metallic layers as well. For example, in nanostripe systems^20^,^21^, as the top antenna couples incident light into SP waves propagating beneath, both the absorptions of the top stripe and the bottom Au film are improved. The consequence is, although the resonant total absorption is nearly perfect, the net absorption is not necessarily high.

In this study, we design a plasmonic hot-electron photodetection system under conformal metal-semiconductor-metal (MSM) grating configuration, which is a modified MIM structure and shows no qualitative difference in underlying operation principles with those reported in Refs 18,21 since doping is not needed for the proposed device. This is substantially different from the conventional MS systems based on Schottky junctions composed by nanostructured metal film and heavily doped semiconductors^20^. The replacement of insulator by the undoped wide-bandgap semiconductor facilitates the electron transport through the barrier and the conformal MSM photonic setup enables intensified SPs and a strongly asymmetric optical absorption, bridging a feasible way to dramatically improve the optical performance and photoresponsivity^24^. Based on full-wave finite-element electromagnetic simulation and analytical probability-based electrical calculation, the detailed optical and electrical responses of the conformal MSM photodetector are evaluated. The simulated results demonstrate a highly asymmetrical optical absorption, i.e., over 99% of the incident light is absorbed by the top metallic grating, leading to a high and unidirectional photocurrent flow. Our electrical prediction shows that the SPs-enhanced photoresponsivity can be up to 0.032 mA/W without an external electrical bias. Moreover, the proposed conformal system has a resonance with high tunability and sensitivity [i.e., a full width at half maximum (FWHM) of ~10 nm in this study] for photodetection in various spectral bands.

The schematic of the proposed conformal MSM photodetector is shown in Fig. 1(a), which is composed of the silica substrate, the bottom gold (Au) grating, the middle zinc oxide (ZnO) semiconductor layer, and the top conformal Au layer. The width, height and period of the bottom grating are denoted as *w*, *d*_3_ and *Λ*, respectively. *d*_1_ (*d*_2_) is the thickness of the top Au (ZnO) layer. In our calculation, the transverse magnetic (TM, i.e., magnetic field along the grating stripes) light is projected normally onto the system, in order to excite SPs from the metallic grating. Figure 1(b) shows the energy diagram of Au-ZnO-Au junction under a bias of *V*_b_. Once the SPs are excited, the incident light is efficiently and preferentially coupled into the plasmonic system, pumping the free electrons up to a higher energy state above the Fermi level of the metal. If the energy of the excited electron exceeds the barrier potential (*Φ*_b_), the corresponding hot electron will diffuse to the MS interface, tunnel through the semiconductor layer, and finally be collected by the bottom metal.

The proposed conformal MSM hot-electron photodetector is designed and evaluated from both optical and electrical perspectives. The optical description is based on the electromagnetic model, which can be calculated by using full-wave finite-element method^25^. The electrical treatment addresses the detailed processes of the generation and transport of hot electrons^21^,^26^. More information on the numerical method can be found in the supplementary information.

To obtain high photoresponsivity, the well-controlled optical response plays a crucial role. First, the strongly coupled and intensified plasmonic resonance has to be excited through the conformal/multilayered metallic grating system. The detection wavelength (λ_spp_) under SP resonance is mainly determined by the grating period (*Λ*) according to the phase-matching condition^8^.


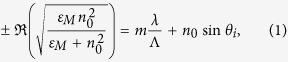


where 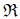 is the operator of real part, *m* the order of diffracted waves, *ε*_M_ the dielectric constant of Au, *n*_0_ the refractive index of the environment (air here), and *θ*_i_ the incident angle. However, even the presence of the excited SPs leads to strong system absorption, a high photocurrent cannot be guaranteed since it is subject to the highly asymmetrical optical absorption. This requires a thorough device optimization in order to minimize the absorption of the bottom Au layer and simultaneously maximize the absorption of the top Au layer.

Plotted in Fig. 2 are the complete dispersion diagrams of the resonances of the conformal plasmonic system with respect to the key device parameters, including the grating period *Λ*, the bottom grating width *w*, the top grating thickness *d*_1_, and the bottom grating thickness *d*_3_. In this figure, *A*_net_ (defined as the top metal absorption minus the bottom metal absorption) is used to evaluate the optical performance of the system. It should be mentioned that, the thickness of the semiconductor layer *d*_2_ = 4 nm is used throughout this study to enable the generated hot electrons tunneling through the dielectric layer with a high probability. Figure 2(a) shows that: 1) the conformal MSM system under plasmonic resonance can absorb almost the entire incident energy; 2) *λ*_spp_ is linearly proportional to *Λ* when *Λ* > 560 nm, as predicted by Eq. (1); 3) dispersion property exhibits quite abnormal behavior when *Λ* < 560 nm due to the weak plasmonic in-coupling efficiency under a high fill factor (~95%, defined as the ratio of the grating width to the grating period) of metallic material. Adjusting the top grating thickness *d*_1_ brings un-noticeable change to *λ*_spp_ but a substantial effect on *A*_net_ [see Fig. 2(b)]. The inset of Fig. 2(b) indicates that if *d*_1_ is too small, *A*_net_ becomes negative, revealing that the bottom metal absorption dominates the total absorption. Although it seems that a larger *d*_1_ is beneficial for a higher *A*_net_, from the electrical viewpoint the top metal should not be too thick in order not to degrade the transport efficiency of the excited hot electrons towards the MS interface. Therefore, *d*_1_ should be properly selected to balance the light absorption and electron collection. Comparably, *d*_3_ and *w* influence the resonance in a more dramatic way with apparent optimal ranges for the optimization of *A*_net_ [Fig. 2(c,d)]. The high tunability through *d*_3_ indicates that, although the bottom Au grating does not seem to absorb the incident energy, it strongly affects the resonant wavelength and the absorption peak. Therefore, one of the functionalities of the bottom metal layer is to provide a convenient way to control and optimize the response of the MSM photodetection system.

Once strong and highly asymmetrical optical absorption is achieved, we need to address the electrical response in detail in order to obtain a thorough evaluation of the proposed device. In this study, we present both optical [Fig. 3(a)] and electrical [Fig. 3(b)] comparisons for systems based on conformal and conventional grating setups [see the insets of Fig. 3(b)]. It should be noted that the electrical simulation for the conformal system becomes much more complicated in order to accurately model the electron transport in the multi-section cavities. Here we just present the simulation results, and the detailed numerical treatment is provided in the supplementary material.

For a fair comparison, both conformal and conventional setups are configured to support plasmonic resonance at *λ*_spp_ = 682 nm under an identical grating period. The detailed device parameters have been given in the figure caption. Figure 3(a) plots the absorption spectra of the top and bottom metal layers for both systems. It is obvious that the conformal one exhibits an extremely higher (lower) top (bottom) metal absorption, i.e., >99% (<1%), leading to a net absorption over 98%; nevertheless, under the conventional design the absorption of the top metal layer (A_top_) is only around 50%. It should be indicated that the bottom metal absorption in the conventional system is around 12% as shown in Fig. 3(a), instead of approaching 50% (i.e., 1 – A_top_ = 50%). This is because only the part of the bottom metal layer directly beneath the top grating has been considered (the absorbed energy by the lateral parts contribute negligibly to the photocurrent)^21^. The huge difference in the net optical absorption (98% versus 38%) exhibits the apparent benefit of using a conformal setup for hot-electron photodetection, which requires a strongly unidirectional photocurrent from an asymmetrical optical absorption. Such an outstanding optical property for plasmonic hot-electron photodetection has seldom been reported. As expected from the optical response, under plasmonic resonance the conformal MSM structure has a much higher photoresponsivity (~0.032 mA/W) than the conventional one (~0.012 mA/W) [Fig. 3(b)]. Another important observation is the narrow-band response. According to our calculation, the FWHM of the conformal MSM design is ~10 nm. Such a sharp resonance can find quite diverse applications, including high-sensitivity and narrow-band detection, sensing, etc. Compared to the conventional metal-semiconductor systems^20^, the introduction of the bottom metal film yields a much narrower resonant spectrum with a much higher absorption peak, due to the hybrid nature of the resonant modes excited from the MSM structure.

The underlying physics behind the dramatic performance improvement lies in the substantially enhanced net absorption as well as a higher transport probability for the hot electrons. To better understand these mechanisms, Fig. 4 illustrates the spatial distributions of the absorbed power in the conformal (left column) and conventional (right column) MSM structures with the device parameters taken from Fig. 3. As shown in Fig. 4(a,b), under blue-shifted off-resonant incidence, both systems exhibit similar behavior, i.e., top metal grating dominates the moderate optical absorption. On resonance, the excitation of SPs boosts dramatically the light absorption; however, the spatially localized absorption is quite distinct between conformal and conventional systems. For the latter, both the top and the bottom metal layers strongly absorb the light [Fig. 4(d)]; nevertheless, the bottom metal layer shows almost no optical absorption in the conformal system [Fig. 4(c)]. This sharp difference originates from the distinct system configurations, i.e., the bottom metal does not directly interact with the incident light in the conformal system, unlike that happened in the conventional system, where there is a strong coupling between the incidence and the bottom metal. Therefore, the conformal design can not only guarantee highly tunable and intensified SPs to be excited with a high efficiency, but also minimize the bottom metallic layer absorption, leading to high unidirectional photocurrent. Under red-shifted incidence, the long-wavelength light is absorbed mostly by the corners of the plasmonic system [Fig. 4(e,f)]. By considering the electron collection capability, the absorption patterns under resonance [Fig. 4(c,d)] show that most of the hot electrons are generated in the regions close to the top MS interface, facilitating electron diffusion towards the opposite electrical terminal.

Finally, we perform a comparison on the response of the conformal MSM photodetection system under TM and TE (transverse electric) illuminations in order to validate the role of plasmonics in enhancing the photodetection sensitivity. Results are plotted in Fig. 5(a), which indicates that the system under TE incidence shows no peaks and the absorption in the whole band is extremely low (in this case the device is highly reflective); on the contrary, TM incidence leads to almost perfect absorption (100%) under plasmonic resonance, showing that plasmonics is a promising way to boost the hot-electron photodetection sensitivity. As most electronic devices, applying an electric bias can further improve the electrical performance of the device since it modifies the internal potential distribution and therefore dramatically affects the carrier transportation (recombination). In this study, we apply both negative and positive electric biases to the photodetector and examine the variation of the device responsivity against the light injection. Our results are shown in Fig. 5(b), which indicates that the forward bias can further enhance the responsivity, but the negative one greatly degrades the system performance. This is because the forward (negative) electric bias leads to decreased (increased) barrier, allowing a higher (lower) hot-electron transmission probability.

In summary, we have presented the design of a plasmonic hot-electron photodetector, which supports strong and highly asymmetrical optical absorption with high tunability, by introducing conformal MSM grating configuration. The optical response and hot-electron generation were evaluated by electromagnetic calculation. The detailed electrical simulation was used to quantitatively characterize the device photocurrent. Compared with a conventional grating MIM configuration, the conformal system provides a net absorption over 98% (38% for conventional one). Correspondingly, our electrical simulation predicts that the unbiased photoresponsivity of the device is ~0.032 mA/W, which is ~3 times of that based on a conventional grating MIM design. We also examined the device response under TE incidence and observed an extremely low absorption, confirming the crucial role of SPs in improving the photoresponsivity. The new architecture of conformal MSM grating structure shows a great potential as an alternative candidate for applications in photodetection and energy conversion.

## Fabrication suggestion

**Figure 6:**
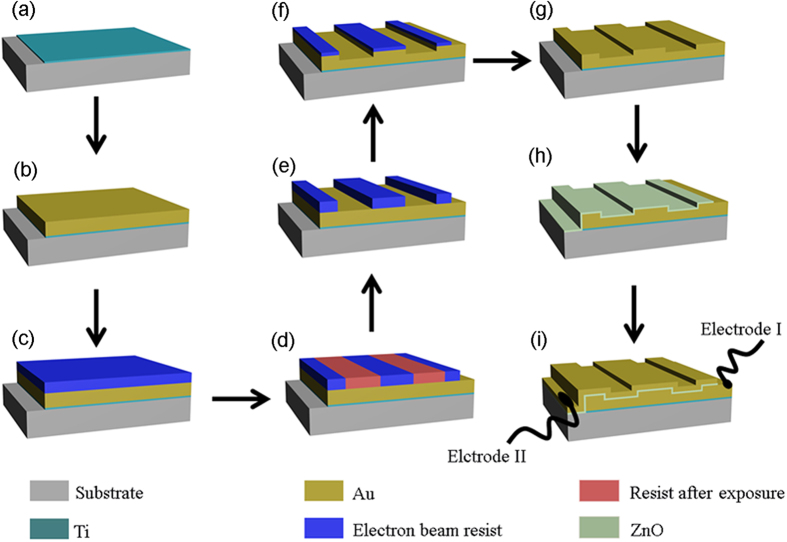
Suggested fabrication procedure of the conformal MSM hot-electron photodetection device.

A suggested fabrication process for the proposed conformal MSM hot-electron photodetector is shown in Fig. 6. First, a thin Ti adhesion layer is deposited by electron-beam evaporator on a quartz substrate [Fig. 6(a)]. Then, a thick Au film is evaporated onto the adhesion layer [(Fig. 6(b)]. Electron beam resist is spin-coated and exposed by electron beam lithography technology [(Fig. 6(c,d)]. 1-D aligned stripes are obtained after lifting-off the exposed resist [(Fig. 6(e)]. Ar ion beam is employed to etch the resist-patterned sample, and the patterns can be copied to the Au film after removing the residual resist [(Fig. 6(f,g)]. Next, 4-nm-thick ZnO film and a thin Au film are deposited in sequence by atomic layer deposition (ALD) in order to guarantee the good conformal morphologies [(Fig. 6(h,i)]. Finally, two electrodes/probes contacting separately the top and bottom Au films are created to enable current-voltage measurements.

## Additional Information

**How to cite this article**: Wu, K. *et al.* Strong and highly asymmetrical optical absorption in conformal metal-semiconductor-metal grating system for plasmonic hot-electron photodetection application. *Sci. Rep.* **5**, 14304; doi: 10.1038/srep14304 (2015).

## Supplementary Material

Supplementary Information

## Figures and Tables

**Figure 1:**
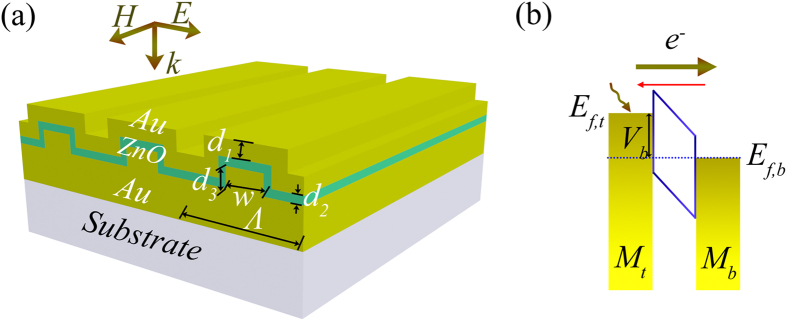
Schematic configuration and operation principle of the proposed conformal MSM hot-electron photodetection device. (**a**) Schematic diagram of the MSM photodetector with hot electrons excited by using conformal metallic grating. (**b**) Energy band diagram of Au-ZnO-Au junction under a bias of *V*_b_. Hot electrons excited in the top (bottom) metallic layer diffuse towards the MS interface and tunnel through the semiconductor layer to the bottom (top), generating an upward (downward) photocurrent.

**Figure 2:**
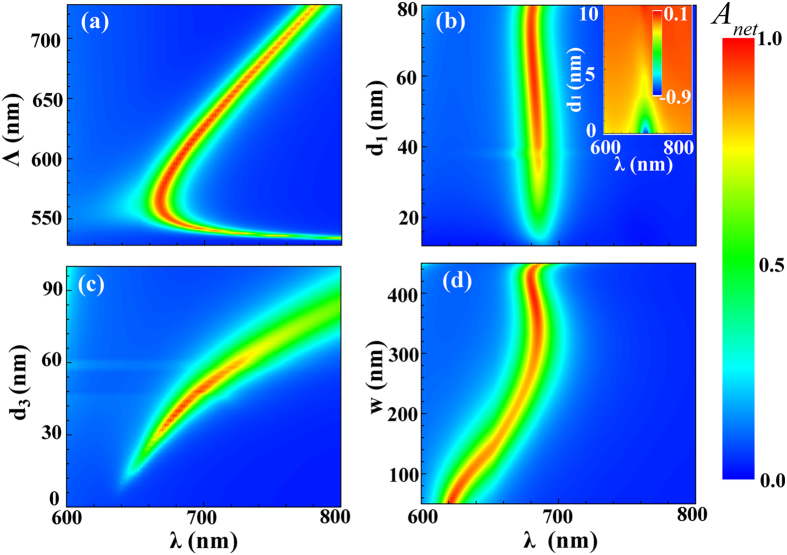
Tunability of the optical resonance. The net absorption spectra as function of *Λ* (**a**), *d*_1_ (**b**), *d*_3_ (**c)**, and *w* (**d**). In the simulations, *Λ* = 600 nm, *w* = 400 nm, *d*_1_ = 60 nm, *d*_2_ = 4 nm, and *d*_3_ = 40 nm, unless otherwise specified.

**Figure 3:**
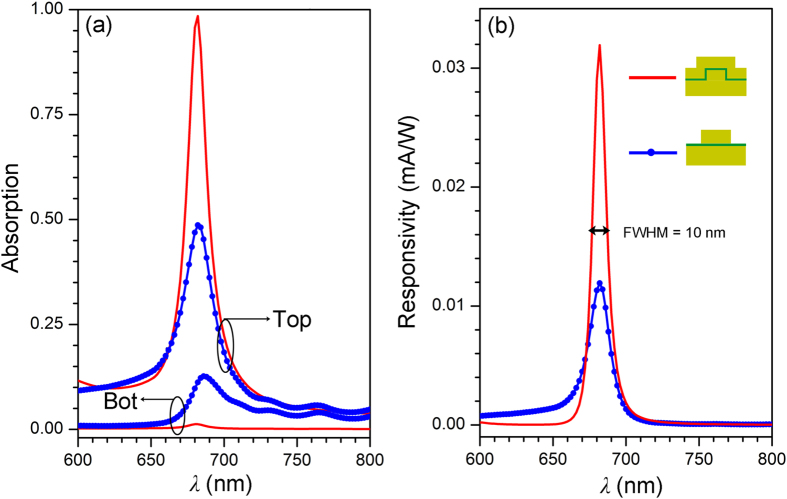
Absorption and responsivity spectra. Absorption (**a**) and responsivity (**b**) spectra of the conventional and the conformal grating systems. Both systems are designed to support plasmonic resonance under an identical wavelength (*λ*_spp_ = 682 nm). For the conformal system: *Λ* = 600 nm, *d*_1_ = 60 nm, *d*_2_ = 4 nm, *d*_3_ = 40 nm, and *w* = 400 nm; for the conventional system: *Λ* = 600 nm, *d*_1_ = 40 nm, *d*_2_ = 4 nm, and *w* = 420 nm (here *Λ* is the grating period, *d*_1_ the top grating thickness, *d*_2_ the ZnO thickness, and *w* the top grating width).

**Figure 4:**
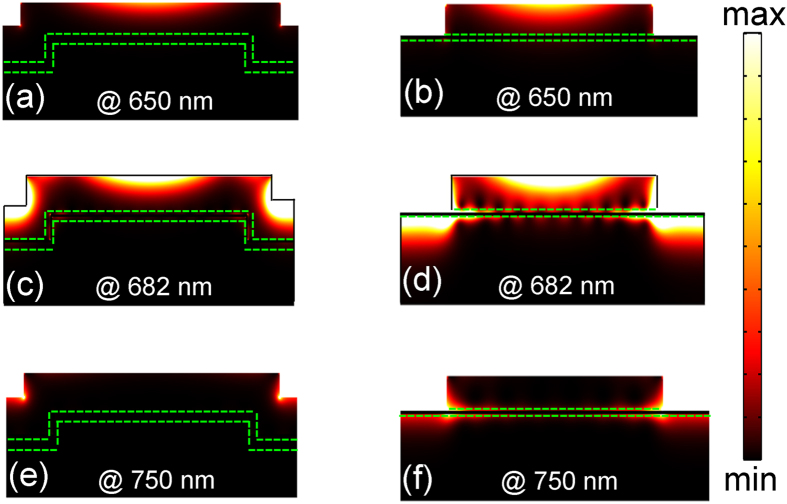
Power absorption patterns. The spatial distributions of the power absorption in the conformal (left column) and conventional (right column) MSM grating hot-electron photodetection devices. The wavelengths under considerations have been inserted in the figures.

**Figure 5:**
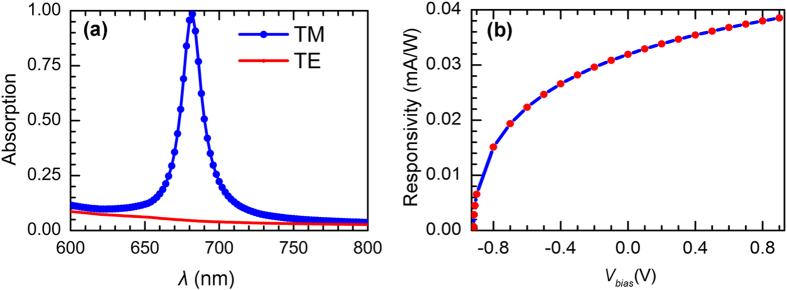
Absorption spectra and responsivity versus applied bias. (**a**) Absorption spectra of the conformal MSM hot-electron photodetector under TM (blue) and TE (red) incidences. (**b**) Responsivity as function of the applied bias. The device parameters are from the previous optimization, i.e., *Λ* = 600 nm, *d*_1_ = 60 nm, *d*_2_ = 4 nm, *d*_3_ = 40 nm, and *w* = 400 nm.
