# Supplementary material for: Strong and highly asymmetrical optical absorption in conformal metal-semiconductor-metal grating system for plasmonic hot-electron photodetection application
Source: Sci Rep. 2015 Sep 21;5:14304. doi: 10.1038/srep14304 (PMC4585709; doi:10.1038/srep14304)
Supplement: Supplementary Information [file srep14304-s1.pdf]

Strong and highly asymmetrical optical absorption in conformal metal-semiconductor-metal grating system for plasmonic hot-electron photodetection application –Supplementary information

Kai Wu[1,2], Yaohui Zhan[1,2], Cheng Zhang[1,2], Shaolong Wu[1,2], and Xiaofeng Li[1,2,*]

[1]College of Physics, Optoelectronics and Energy & Collaborative Innovation Center of Suzhou Nano Science and Technology, Soochow University, Suzhou 215006, China.

[2]Key Lab of Advanced Optical Manufacturing Technologies of Jiangsu Province & Key Lab of Modern Optical Technologies of Education Ministry of China, Soochow University, Suzhou 215006, China.

Corresponding author: xfli@suda.edu.cn

Detailed optical and electrical calculation

The whole transport process from light absorption to photocurrent output can be sequenced into the following three steps.

Step 1: light absorption with the absorptance (A) defined as $A = A_{\mathrm{abs}}/A_{\mathrm{inc}}$, where A_{abs} (A_{inc}) is the absorbed (incident) power. A_{abs} can be obtained by

$$A_{abs}(\boldsymbol{r},\omega) = \frac{1}{2}\omega Im(\varepsilon_M)|E(\boldsymbol{r},\omega)|^2 \quad \text{(S1)}$$

where $\boldsymbol{r}$ is the spatial variable, ω the angular frequency, $Im(\varepsilon_{\mathrm{M}})$ the imaginary part of dielectric function of Au[1], and $|\boldsymbol{E}|$ the electric field.

Step 2 corresponds to the process that some of excited hot electrons diffuse to the Au/ZnO interface without losing energy in inelastic collision. In this part the electron mean free path (MFP) is considered in the calculation of the average probability P_{i}

$$P_i = \frac{\sum_j^m \exp\left(\frac{-y_i}{L_e}\right)}{m} \cdot \frac{\theta_i}{2\pi}, \quad \text{(S2)}$$

where i denotes the ith electron, y_{i} (θ_{i}) is the diffusion distance (angle) of this electron to the M/S interface which has been discretized into m sections, and L_{e} is the electron mean free path (MFP) in gold (70 ~ 20 nm in the energy range of 1 ~ 2 eV[2]).

The numbers of the generated hot electrons that reach the M/S interface is calculated by

$$N_i = \frac{Q_i}{h\nu} \cdot P_i, \quad \text{(S3)}$$

where Q_{i} is the absorbed energy, h the Plank's constant, and ν the optical frequency. The total numbers of hot electrons generated in the top metal that reach the M/S interface can be expressed as

$$N_{Tot} = \sum_{i=1}^{n} N_i. \quad \text{(S4)}$$

Step 3 is a quantum process where the hot electrons tunnel through M/S interface to the opposite metallic layer and finally generate photocurrent. This part also can be further categorized into three sub-steps: i) the electrons tunnel through the ultra-thin barrier; ii) the hot electrons pass through the oxide layer without inelastic collisions; iii) hot electrons arrive at the ZnO/Au interface

and transmit into the bottom Au layer. In step i), the barrier height is related to the work function (W) of the metal and the electron affinity (ϕ) of the semiconductor, so the variation of materials selected for photodetection in the specific band leads to quite unique optical (especially electrical) response, which has to be carefully addressed in the design of the hot-electron photodetection system; for Au, W_{Au} = 5.1 eV[3], and for ZnO, ϕ_{ZnO} = 4.2 eV[4], so the barrier energy Φ_b = 0.9 eV. There are many models that describe this quantum process[5-8], among which the most widely adopted is the WKB approximation which assumes that the transmission probability is close to 100% when the electron energy (E_{ph}) > Φ_b; otherwise, the probability is close to zero. However this model is too simplified without considering sufficient physical processes, such as the electron reflections between the interfaces of two different materials as described in Scals's model. Therefore, Chalabi's model is used in our calculation which considers the hot electron's reflection process and has been proved to be accurate enough to show a good consistence with experiment. In step ii), the probability of hot electrons transportation can be estimated by

$$P = \exp\left(-d_{\mathrm{oxide}}/\lambda_{\mathrm{MFP\_ox}}\right), \tag{S5}$$

where d_{oxide} is the thickness of the oxide layer and $\lambda_{\mathrm{MFP\_ox}}$ is the mean free path (MFP) in ZnO. MFP is energy-dependent with range of 10 ~ 30 nm for the energy of 1 ~ 2 eV[9]. In our study, 20 nm is a reasonable value to estimate the quantum efficiency. So probability of this process is relative large (~ 0.8). Step iii) describes the process that hot electrons arrive at the ZnO/Au interface and transmit into the bottom Au layer which is similar to step i).

The hot electrons generated in the bottom metal layer also obey the same three sub-step process, but forming the downward photocurrent. In our calculation, the energy distribution of hot electrons above Fermi level can be approximated to be uniform. Therefore, the total current can be written as

$$I_{Net} = I_{Top\to Bot} + I_{Bot\to Top} = \frac{1}{E_{ph}} \iint_{\Phi_b}^{E_{ph}} q\left(N_{Top}P_{Top} - N_{Bot}P_{Bot}\right) dE, \tag{S6}$$

where N_{Top} and N_{Bot} are the total numbers of hot electrons which are excited by the top and bottom metal layers. I_{Net} is the net photocurrent, I_{Top} (I_{Bot}) the downwards (upwards) photocurrents, q the elemental charge, and P_{Top} (P_{Bot}) the corresponding total transmission probability of the excited hot electrons.

Compared to the conventional grating system [Fig. S1(a)], the electron transportation and collection process in the proposed conformal system [Fig. S1(c)] become much more complicated. Considering that the generated hot electrons diffuse isotropically in metal and those electrons that never reach the M/S interface do not contribute detectable photocurrent, the average probabilities (P_{i}) of the hot electrons arriving at the M/S interface have to be used. As shown in Figs. S1(a) and S1(c), for the top metal absorption, only the hot electrons confined within the angle θ [shown in Figs. S1(a) and S1(b)] can be possible to successfully diffuse to the M/S interface (it is similar for those electrons generated in the bottom metal layer). We divide the top grating layer into several sections for the ease of calculation. Shown in Figs. S1(b) and S1(d) are the calculated average probability (P_{i}) for different parts (I, II and III). It is observed that the hot electrons can move more easily to the M/S interface in conformal structure under a larger confinement angle (θ) and a shorter electron transportation path.

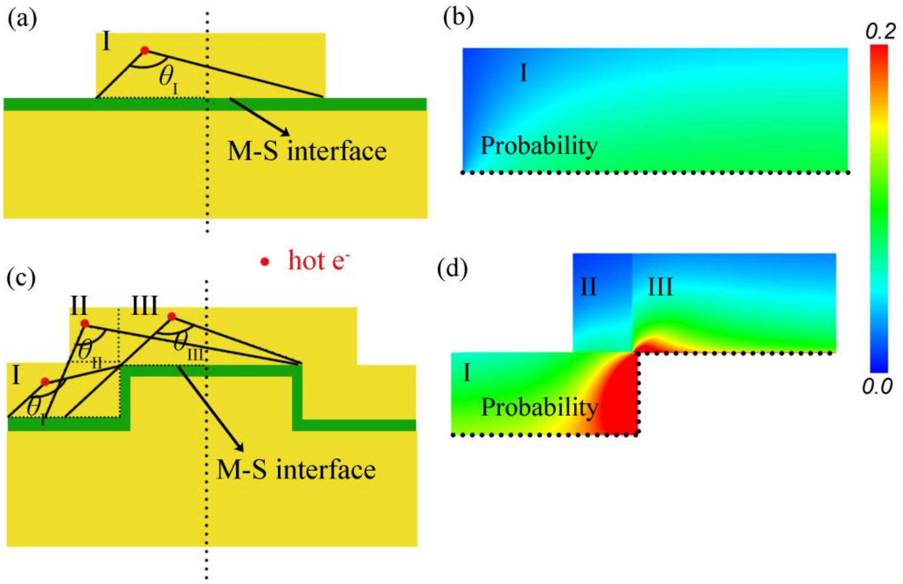

Figure S1. Detailed treatment on the diffusions of hot electrons in the top metallic layer in conventional (a) and conformal (c) MSM structures. Spatial distributions of the probabilities for step 2 (electron diffusion to M/S interface) in the conventional (b) and conformal (d) cases.

References

1. Palik, E. D. *Handbook of optical constants of solids.* (Academic Press: Orlando, 1985).
2. Sze, S. M., Moll, J. L. & Sugano, T. Range-energy relation of hot electrons in gold. *Solid. State. Electron*. **7**, 509–523 (1964).
3. Michaelson, H. B. The work function of the elements and its periodicity. *J. Appl. Phys*. **48**, 4729–4733 (1977).
4. Brillson, L. J. & Lu, Y. ZnO schottky barriers and ohmic contacts. *J. Appl. Phys.* **109**, 121301 (2011).
5. Stuart, R., Wooten, F. & Spicer, W. E. Monte Carlo calculations pertaining to the transport of hot electrons in metals. *Phys. Rev*. **135**, A495–A505 (1964).
6. Fowler. R. H. The analysis of photoelectric sensitivity curves for clean metals at various temperatures. *Phys. Rev*. **38**, 45–46 (1931).
7. Gundlach, K. H. Theory of metal-insulator-metal tunneling for a simple two-band model. *J. Appl. Phys*. **44**, 5005–5010 (1973).
8. Kovacs, D. A., Winter, J., Meyer, S., Wucher, A. & Diesing, D. Photo and particle induced transport of excited carriers in thin film tunnel junctions. *Phys. Rev. B*. **76**, 235408 (2007).
9. Seah, M. P. & Dench, W. A. Quantitative electron spectroscopy of surfaces: a standard data base for electron inelastic mean free paths in solids. *Surf. Interface Anal*. **1**, 2–11 (1979).
